# Phylogeny, divergence time and historical biogeography of *Laetiporus* (Basidiomycota, Polyporales)

**DOI:** 10.1186/s12862-017-0948-5

**Published:** 2017-04-20

**Authors:** Jie Song, Bao-Kai Cui

**Affiliations:** Institute of Microbiology, Beijing Forestry University, P.O. Box 61, 35#, Qinghua East Road, Haidian District, Beijing, 100083 People’s Republic of China

**Keywords:** *Laetiporus*, Wood rot fungi, Phylogeny, Biogeography, Molecular clock

## Abstract

**Background:**

The aim of this study was to characterize the molecular relationship, origin and historical biogeography of the species in important brown rot fungal genus *Laetiporus* from East Asia, Europe, Pan-America, Hawaii and South Africa. We used six genetic markers to estimate a genus-level phylogeny including (1) the internal transcribed spacer (ITS), (2) nuclear large subunit rDNA (nrLSU), (3) nuclear small subunit rDNA (nrSSU), (4) translation elongation factor 1-α (EF-1α), (5) DNA-directed RNA polymerase II subunit 2 (RPB2), and (6) mitochondrial small subunit rDNA (mtSSU).

**Results:**

Results of multi-locus phylogenetic analyses show clade support for at least seventeen species-level lineages including two new *Laetiporus* in China. Molecular dating using BEAST estimated the present crown group diverged approximately 20.16 million years ago (Mya) in the early Miocene. Biogeographic analyses using RASP indicated that *Laetiporus* most likely originated in temperate zones with East Asia and North America having the highest probability (48%) of being the ancestral area.

**Conclusions:**

Four intercontinental dispersal routes and a possible concealed dispersal route were established for the first time.

**Electronic supplementary material:**

The online version of this article (doi:10.1186/s12862-017-0948-5) contains supplementary material, which is available to authorized users.

## Background

Since the late Tertiary period, severe climatic change and major geological events have played important roles in driving species diversity and in shaping the biogeographic distribution of extant organisms. Benefiting from the development of DNA technology and molecular analysis methods, studies of fungal molecular phylogeny and biogeography have been conducted in recent decades [1–3]. Based on molecular dating, many phylogenetic studies have revealed striking chronological and geographical correlations between evolutionary divergence and geological events [3–8].

*Laetiporus* Murrill (Fomitopsidaceae, Polyporales) is a cosmopolitan genus, typified by *L. sulphureus* (Bull.) Murrill [9]. Species in this genus grow from cold temperate to tropical zones and are associated with Betulaceae, Burseraceae, Elaeocarpaceae, Fabaceae, Fagaceae, Meliaceae, Myrtaceae, Oleaceae, Pinaceae, Salicaceae, Sapindaceae and Taxaceae [10–15]. *Laetiporus* spp. have been considered to be forest pathogens and to cause brown cubical heart rot [16, 17], which is implicated in the cycle of the forest ecosystem [13, 15]. Chemical composition research determined that this cultivable mushroom is a potential food due to its rich digestible bioactive substances and lack of detectable levels of poisonous microelements [18]. Some taxa of *Laetiporus* are also valuable sources of medicine, such as ergosterol and acetyl eburicoic acid [19, 20].

Recently, several studies were carried out to clarify the species diversity and phylogeny of *Laetiporus* [11–15]. In these studies, six new species were described, and four new lineages were identified: Clade I, Clade H, Clade L and Clade M. In addition, *L. sulphureus* and *L. versisporus* (Lloyd) Imazeki were shown to each be divisible into three different lineages [11–15], which are represented here as Clade C, Clade E1/E2 and Clade G1/G2/G3, respectively.

To date, eleven species and four undescribed taxa of *Laetiporus* have been accepted as belonging to this genus [15]: *L. ailaoshanensis* B.K. Cui & J. Song, *L. cremeiporus* Y. Ota & T. Hatt., *L. versisporus* and *L. zonatus* B.K. Cui & J. Song from East Asia; *L. cincinnatus* (Morgan) Burds., Banik & T.J. Volk, *L. conifericola* Burds. & Banik and *L. huroniensis* Burds. & Banik from North America; *L. caribensis* Banik & D.L. Lindner from Central America; *L. montanus* Černý ex Tomšovský & Jankovský from East Asia and Europe; *L. sulphureus* from North America, South America and Europe; *L. gilbertsonii* Burds. from Pan-America; *L.* sp. 1 from Hawaii; *L.* sp. 2 from South America; *L.* sp. 3 and *L.* sp. 4 from Central America [11–15]. However, the interspecies relationships within *Laetiporus*, as well as the origin and biogeography of the genus, remain unclear.

Here, we present multi-locus phylogenetic analyses using sequences from the internal transcribed spacer (ITS), nuclear large subunit rDNA (nrLSU), nuclear small subunit rDNA (nrSSU), translation elongation factor 1-α (EF-1α), DNA-directed RNA polymerase II subunit 2 (RPB2), and mitochondrial small subunit rDNA (mtSSU) to gain insight into the evolution of species in *Laetiporus*. Our study sought to (1) explore the evolutionary relationships between *Laetiporus* species, and (2) estimate the divergence time and examine hypotheses about the origin and biogeography of *Laetiporus* species.

## Results

### Phylogenetic analyses

**Fig. 1:**
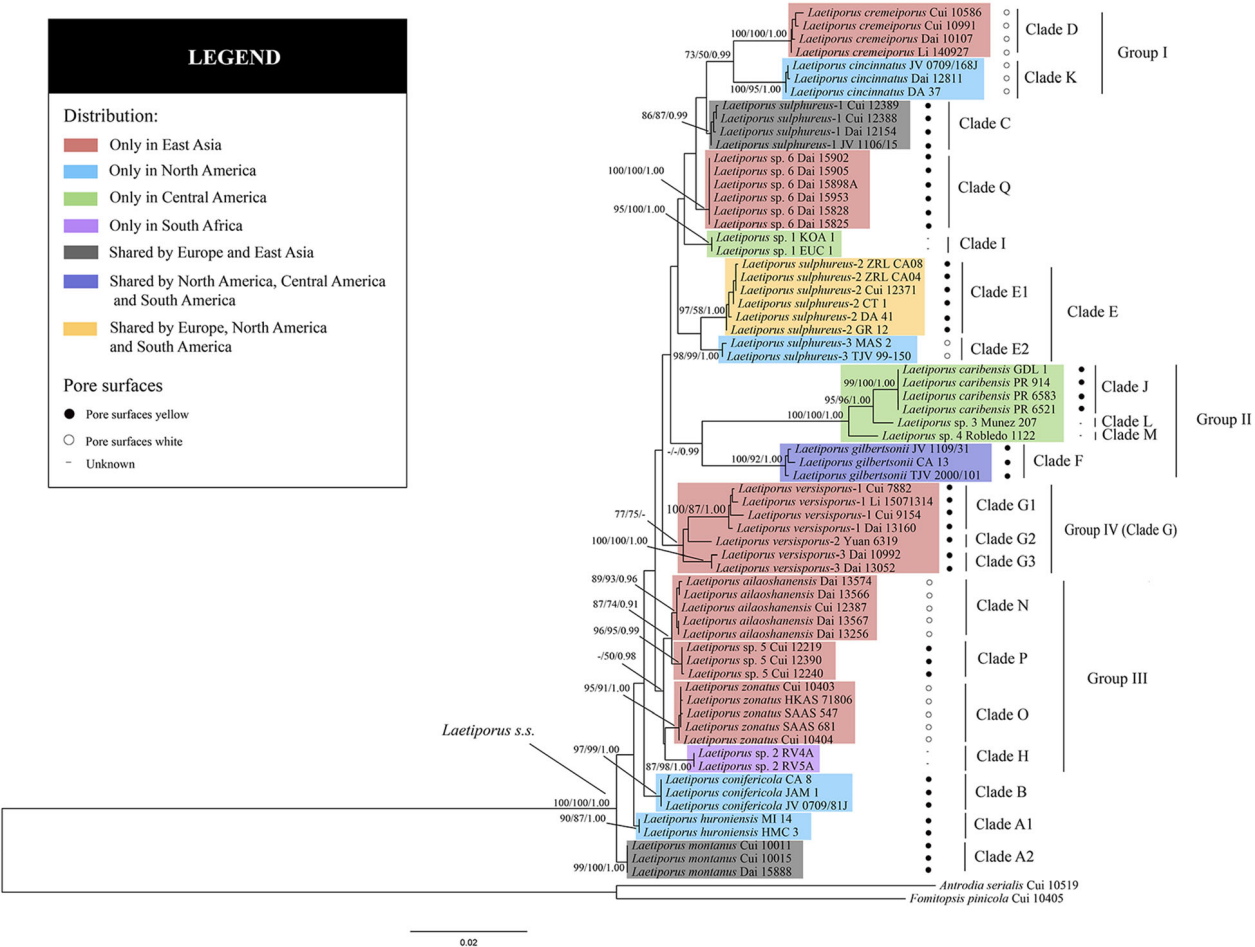
Phylogenetic consensus tree inferred from the maximum likelihood (ML) analysis based on a concatenated, multi-locus dataset (ITS + nrLSU + nrSSU + mtSSU + EF-1α + RPB2). Branches are labeled where MP/BS support is greater than 75% and collapsed below that support threshold. BPP is labeled where greater than 0.90

**Fig. 2:**
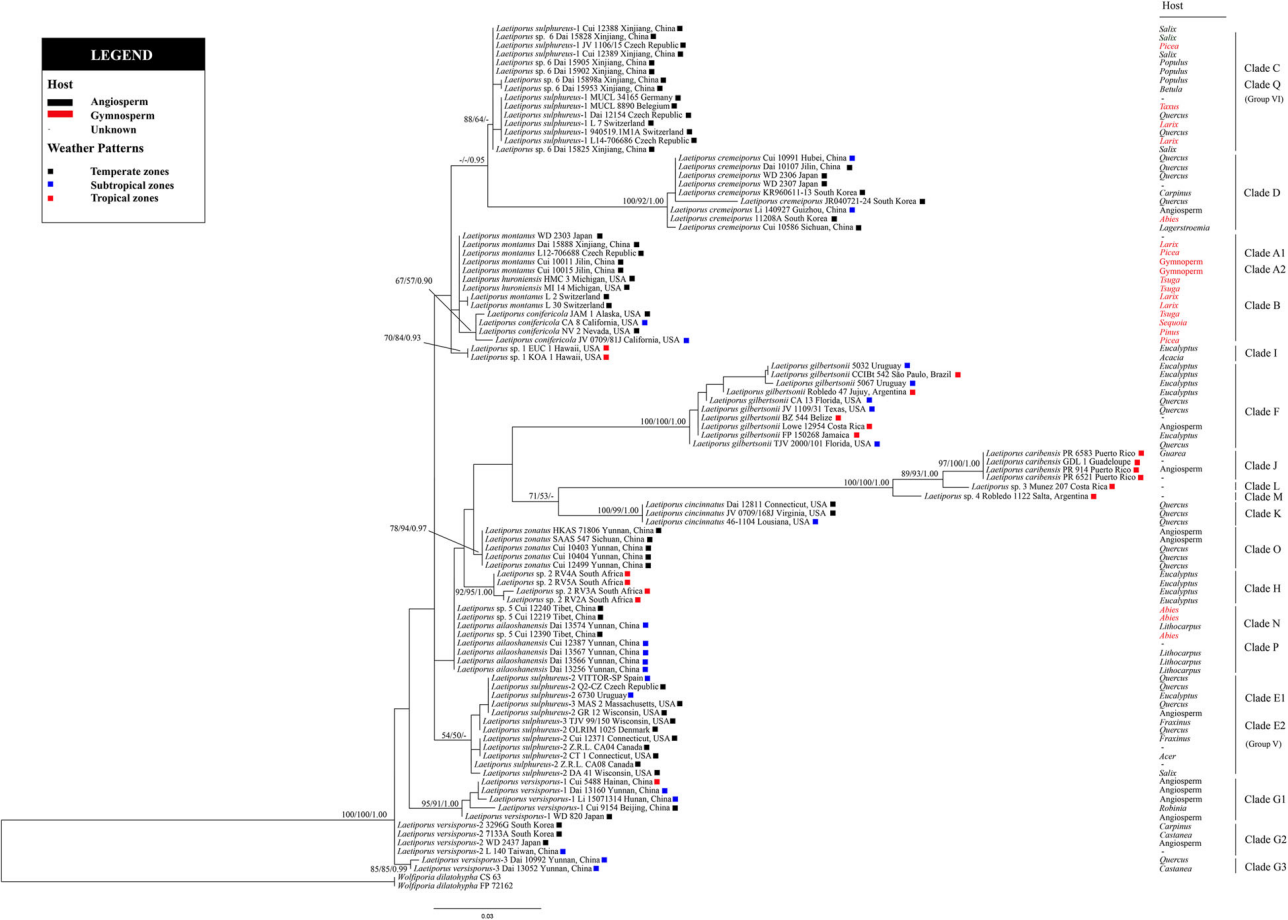
Phylogenetic tree inferred from the maximum likelihood (ML) analysis based on the ITS sequences. Branches are labeled with MP/BS values if greater than 50% and with BPP values if greater than 0.90

The combined dataset (ITS + nrLSU + nrSSU + mtSSU + EF-1α + RPB2) has an aligned length of 3850 characters, of which 3086 are constant, 247 are variable and parsimony uninformative, and 517 are parsimony informative. The tree obtained from the Maximum likelihood (ML) analysis and the maximum parsimony (MP), maximum likelihood (ML) and Bayesian posterior probability (BPP) values based on the dataset are shown in Fig. 1. The aligned ITS matrix comprises 514 positions, of which 378 are constant, 11 are variable and parsimony uninformative, and 125 are parsimony informative. The tree inferred from the ML analysis and the MP, ML and BPP values are shown in Fig. 2.

The combined dataset and ITS dataset inferred similar topologies (Figs. 1 and 2). The genus *Laetiporus* was supported with low levels of support on the stem branches. Moreover, 21 different phylogenetic lineages were inferred and significantly supported by both datasets.

In the combined dataset topology (Fig. 1), *L. sulphureus* was divided into three different well-supported clades: Clade C (86% MP, 87% ML, 0.99 BPP) and Clade E1 (97% MP, 58% ML, 1.00 BPP) with yellow pore surfaces and Clade E2 (98% MP, 99% ML, 1.00 BPP) with a white pore surface. *L. versisporus* was also divided into three different clades: Clade G1 (100% MP, 87% ML, 1.00 BPP), Clade G2 and Clade G3 (100% MP, 100% ML, 1.00 BPP). Three sister lineages (*L. montanus*, *L. huroniensis* and *L. conifericola*) that grow on coniferous trees were well supported (Fig. 1). Two novel phylogenetic species from western China formed two significantly supported terminal lineages and were named Clade P (96% MP, 95% ML, 0.99 BPP) and Clade Q (100% MP, 100% ML, 1.00 BPP). Moreover, four groups were recognized (Fig. 1). Group I is well supported by Bayesian inference (BI) (0.99 BPP) and moderately supported by MP and ML analyses (73% MP, 50% ML), it is composed of two cold-temperate to subtropical *Laetiporus* species with white pore surfaces. Group II is well supported by BI (0.99 BPP) but weakly supported by MP and ML analyses, and it contains four North American, Central American and South American *Laetiporus* species. Group III was well supported by BI (0.98 BPP), moderately supported by ML analysis (50% ML) and includes four *Laetiporus* species with a disjunct distribution from Hengduan-Himalayan zones to South Africa. Group IV was supported by MP and ML analyses (77% MP, 75% ML) and only includes the East Asian species *L. versisporus* with a yellow pore surface.

The ITS dataset (Fig. 2) inferred a similar topology despite some existing differences. Clade E1 and Clade E2 clustered together and formed a novel group (Group V) with moderate support from MP and ML analyses (54% MP; 50% ML) and weak support from BI. Notably, this group was weakly supported by BI, MP and ML in the analyses using the combined dataset. The novel phylogenetic species Clade Q clustered together with Clade C and formed a novel group (Group VI) supported by MP and ML analyses (86% MP; 64% ML) but only weakly supported by BI. Moreover, of the 21 lineages identified in the phylogeny, 14 lineages (67%) have temperate distribution, 9 lineages (43%) have subtropical distribution and 9 lineages (43%) have tropical distribution (Fig. 2).

### Bayesian estimation of divergence time and the historical biogeography of *Laetiporus*

The alignment of the two concatenated datasets (ITS + nrLSU + nrSSU and EF-1α + RPB2), which were 2172 and 1137 bp in length, respectively, consisted of 44 taxa. The aligned ITS dataset was 514 bp in length and was established to estimate the divergence time and biogeographical history of *Laetiporus*.

**Fig. 3:**
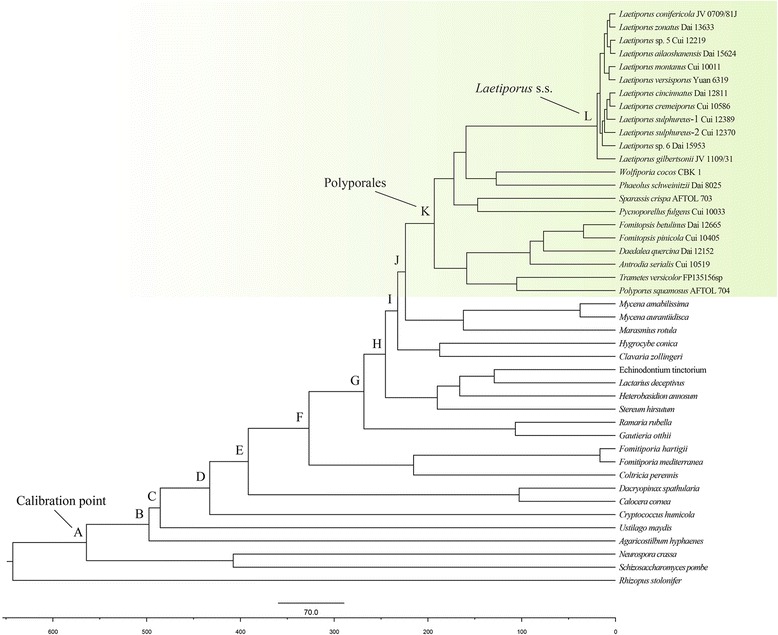
Chronogram and estimated divergence times of *Laetiporus* generated from molecular clock analysis using the ITS + nrLSU + nrSSU and EF-1α + RPB2 datasets. A chronogram obtained using the Ascomycota–Basidiomycota divergence time of 582 Mya as the calibration point is shown. The calibration point and objects of this study are marked in the chronogram. The lineages in the Polyporales are highlighted in green. The geological time scale is in millions of years ago (Mya)

Analyses were calibrated using two methods. First, based on the divergence between Ascomycota and Basidiomycota, at 582 million years ago (Mya), *Paleopyrenomycites devonicus* Taylor, Hass, Kerp, M. Krings & Hanlin (Fig. 3) was used to estimate the divergence time of Polyporales at 194.56 ± 0.89 Mya (141.93–247.52 Mya, 95% higher posterior density (HPD)), which is consistent with a previous inference [21]. The initial diversification of *Laetiporus* occurred during the early Miocene, 20.17 ± 0.12 Mya (12.66–29.09 Mya, 95% HPD), similar to the date of the diversification of the main *Laetiporus* host plants, such as *Quercus*, *Salix*, *Populus*, *Abies*, *Picea* and *Pinus* [1, 4, 22–25]*.* Based on the second calibration point, *Quatsinoporites cranhamii* S.Y. Sm., Currah & Stockey, the divergence between Ascomycota and Basidiomycota was estimated to have occurred at 332.93 ± 3.03 Mya (232.23–447.89 Mya, 95% HPD), which was much more recent than the minimal divergence age of the Ascomycota/Basidiomycota (400 Mya). Meanwhile, the crown age of *Laetiporus* estimated based on the calibration point was approximately 12.26 ± 0.13 Mya (7.04–18.48 Mya, 95% HPD), which was also significantly more recent than is required for the estimated divergence time of the main host plants. Thus, the second calibration point seemed to vastly underestimate the divergence time of *Laetiporus*. Therefore, the first calibration point was used for subsequent analyses, and the divergence times of the main nodes are showed in Fig. 3 and summarized in Additional file 1: Table S1.

**Fig. 4:**
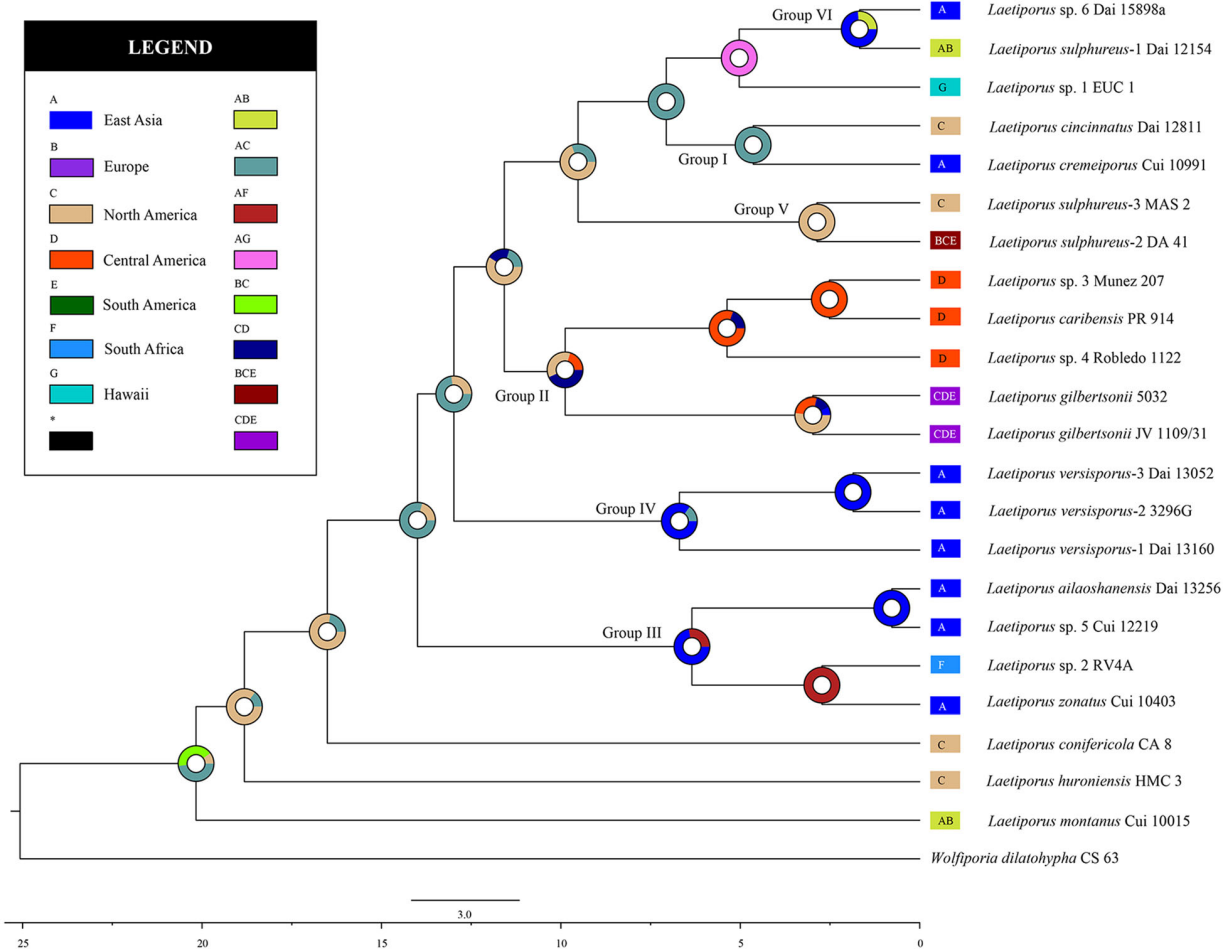
Divergence time estimation and ancestral area reconstruction of *Laetiporus* using the ITS dataset. The chronogram was obtained via molecular clock analysis using BEAST. A pie chart at each node indicates the possible ancestral distributions inferred from dispersal-extinction-cladogenesis (DEC) analysis implemented in RASP. A black asterisk represents other ancestral ranges

**Fig. 5:**
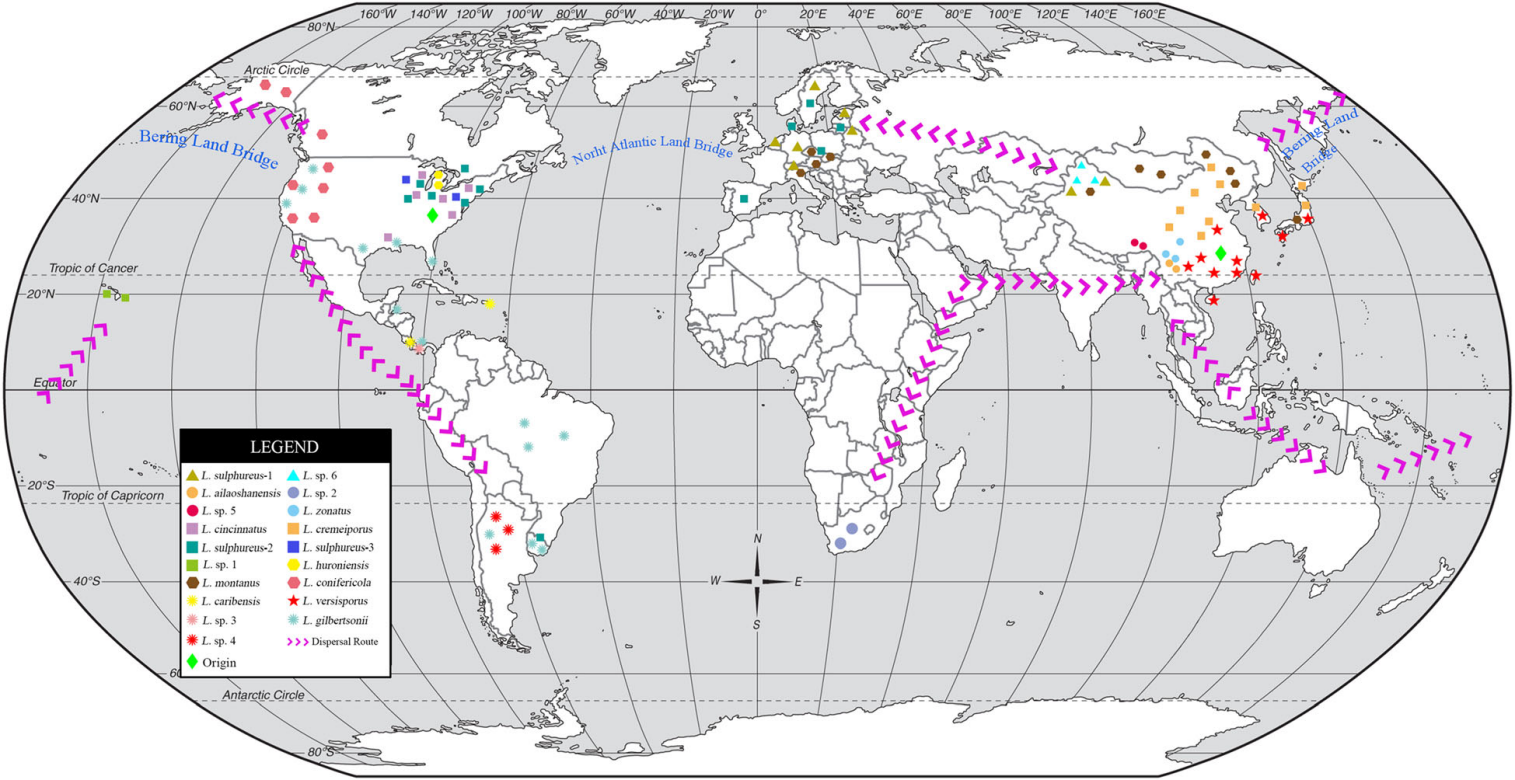
Map of the geographic distribution of *Laetiporus* and possible dispersal routes generated by ArcGIS v10.1. A hypothetical schematic depiction of the original locations, the migration routes, the rapid radiation and the speciation of *Laetiporus*

The inferred historical biogeographic scenarios from analyses using RASP are shown in Fig. 4. The divergence times of the main groups based on the ITS dating analysis are also showed in Fig. 4 and summarized in Additional file 1: Table S3. The results of the Dispersal-Extinction-Cladogenesis (DEC) analysis suggest a complex biogeographic history for *Laetiporus.* Fifteen dispersal events and six vicariance events were necessary to explain the current distribution of the genus. The ancestral area of *Laetiporus* was ambiguous. In the reconstruction of their ancestral geographic range, several areas contribute to the geography in different proportions: the probability for East Asia and North America was 48%, that for Europe and North America was 42%, and that for North America was 10%. Thus, the geographic range of East Asia and North America had the highest probability (48%) of being the ancestral area. The most probable (100%) ancestral area for Group I was East Asia and North America. The most probable (43%) ancestral area for Group II was North America and Central America. East Asia was the most probable ancestral area for Group III and Group IV, at 72% and 85%, respectively. The most probable (100%) ancestral area for Group V was North America. The most probable (74%) ancestral area for Group VI was East Asia. Furthermore, four dispersal routes and a possible concealed dispersal route were inferred: East Asia–eastern North America, North America–Central America–South America, East Asia–South Africa, East Asia–Europe and East Asia–Malay Archipelago–Australia–Hawaii (Fig. 5).

## Discussion

*Laetiporus* has been shown to be a monophyletic group [11–15, 26]. Unexpectedly, despite conducting multi-locus phylogenetic analyses, our study is still unable to entirely resolve the stem relationships within *Laetiporus.* Nevertheless, novel phylogenetic species and certain clustering tendencies are described. Findings regarding the origin, ancestral area and diversification are also inferred.

Group I contains two sister clades, Clade D and Clade K, with disjunct distribution (Fig. 1). Phylogenetically, this group is supported by the combined dataset analyses (73% MP, 50% ML, 0.99 BPP). However, Clade D and Clade K are distant in the ITS topology. Previous studies showed that both species grow on hardwood with the common cool temperate to subtropical habitat, producing an orange pileal surface and cream pore surface (11, 13, 15). The complete gene information, as well as a similar growth habit and morphology between Clade D and Clade K, suggests that the phylogeny inferred from the analyses of the combined dataset is more reliable.

Group II consists of four North/Central/South American *Laetiporus* clades (Fig. 1). Within this group, Clade F is known to reside in temperate to tropical areas with a Pan-American distribution [10, 12, 27]. This distribution indicates a strong adaptive ability. The other three members of *Laetiporus* that behave as sister species are known to reside only in Central America [14], which is part of the Mesoamerican biodiversity hotspot [28]. Species in this group are found on hardwood and share an orange pileal surface and yellow pore surface, although the characters of Clade L and Clade M are uncertain [15]. Notably, their host plants are usually Fagaceae in North America, tropical plants such as *Guarea* and *Dacryodes* in Central America and mainly *Eucalyptus* in South America [11, 14, 27].

Group III contains four *Laetiporus* clades from East Asia and South Africa, including the novel phylogenetic species Clade P (Fig. 1). Clade P is found on *Abies* in cool temperate areas in the Himalayan region. It acts as a sister species with *L. ailaoshanensis* (Fig. 1), which has been found on *Lithocarpus* and *Castanopsis* in subtropical areas in the Hengduan Mountains [15]. Clade O is the other species collected from the Hengduan Mountains, and it grows on *Quercus* in temperate areas [15]. Clade H is found on *Eucalyptus* from South Africa, but its characters remain unclear [12]. The relationships between Clade H and the other three species are uncertain due to the low support in the topology of the combined dataset (Fig. 1). Further studies using samples from South Africa are necessary.

Group IV consists of only *L. versisporus* (Clade G), which has a yellow pore surface (Fig. 1). Previous studies have shown that this species is usually divided into two or three clades [13, 15]. In the current study, *L. versisporus* specimens grouped together with significant support from MP and ML analyses. *L. versisporus* covers most parts of East Asia from the Yunnan-Guizhou Plateau, Hainan to Japan and South Korea, and associate with *Robinia*, *Castanea*, *Quercus*, *Elaeocarpus* and *Castanopsis* [13, 15]. Infraspecific variation and infraspecific hybridization are considered to occur simultaneously [15].

Group V consists of Clade E1 and Clade E2 (Fig. 1). It is obvious that they are closely related and share similar morphology except for the pore surface [11]. Clade E1 is associated with *Quercus*, *Eucalyptus*, *Salix*, *Acer* and *Fraxinus* and has a disjunct temperate to subtropical areas distribution in North America, South America and Europe. Besides, it produces a yellow pore surface [10, 11]. Clade E2 is distributed in temperate areas of North America, is associated with *Quercus* and *Fraxinus*, and produces a white pore surface [10, 11].

Group VI consists of Clade C and the novel phylogenetic species Clade Q (Fig. 2). This group is only supported by the ITS phylogeny, and the phylogeny analyses do not indicate an obvious species boundary. This suggests a close relationship between Clade C and Clade Q. *Laetiporus* Clade C has previously been reported only from Europe [11, 13]. Our study presents the first report of Clade C in Xinjiang, China. This species usually grows on hardwoods and conifers such as *Quercus*, *Sorbus*, *Populus*, *Castanea*, *Prunus*, *Taxus*, *Larix* and *Picea* in temperate areas, producing a yellow pore surface. Clade Q is also found in temperate areas in Xinjiang, China, where it is associated with hardwoods such as *Salix*, *Betula* and *Populus* and produces a yellow pore surface.

The maximum crown age of *Laetiporus* is estimated at the early Miocene (20.17 ± 0.12 Mya) and East Asia and North America are inferred to be the most probable ancestral areas (Figs. 3 and 4). The notable finding is that three coniferous species (*L. montanus*, *L. huroniensis* and *L. conifericola*) in temperate areas behave as sister species in the analyses of the combined dataset (Fig. 1). Moreover, the temperate host plants are diverse, including *Quercus*, *Salix*, *Populus*, *Picea*, *Larix*, *Abies*, *Tsuga*, *Lithocarpus*, *Fraxinus* and *Acer*; in contrast, the tropical host plants are limited in variety, including *Eucalyptus* and *Guarea* [10–15]. Based on these findings, an origin in temperate East Asia and North America is proposed.

The independent sister species in Group I indicate an East Asian–eastern North American dispersal route before the estimated divergence time (4.64 Mya) in the early Pliocene (Fig. 4). This divergence time is close to the break time of the Bering Land Bridge (BLB) at approximately 5.4–5.5 Mya [5]. We speculate that their ancestor covered East Asia and North America via the BLB route and that regional speciation after the vicariance emerged due to the disconnection of the BLB and the severe climate change at that time [29–31]. This route is also present in the dispersal of other organisms, especially the common host plant *Quercus* [1]. There may be a strong dispersal and vicariance correlation between *Laetiporus* spp. and their host plants.

Four *Laetiporus* species in Group II with Pan-American distribution exhibit a North American–Central American–South American dispersal route. This group first diverged at approximately 9.88 Mya. North and Central America are inferred to be the most probable ancestral areas. Clade J, Clade L and Clade M are from Central America and the estimated crown age is approximately 5.38 Mya, which coincides with the paleo-elevations that occurred during the late Miocene and early Pliocene [32]. The second intercontinental distribution between North America and South America is exhibited in Group V (Fig. 4). This route has been confirmed by biogeographical research on plants and animals [1, 33–36]. We speculate that the severe climate change that has occurred since 15 Mya [29] drove the migration from North and Central America to South America and the adaptation to tropical host plants such as *Eucalyptus*, *Guarea* and *Dacryodes*. The vicariance due to tectonic activity is thought to be responsible for the endemism of *Laetiporus* in Central America.

In Group III, four *Laetiporus* species from East Asia and South Africa are closely related (Fig. 1). The estimated divergence time of this group is 6.35 Mya. The DEC analysis inferred East Asia as the most probable ancestral area. However, it is notable that Clade H does not form a robust sister relationship with Clade O (Fig. 1). We speculate that there is incomplete sampling from the Indian Subcontinent to Africa because suitable host plants, such as *Eucalyptus*, are abundant in these areas [12, 37]. Although the estimated divergence time is potentially inaccurate, the dispersal route between East Asia and South Africa is proposed.

The species in Group V also exhibit a continuous distribution in Europe and eastern North America (Fig. 4). The DEC analysis inferred a North American origin for this group, with an estimated divergence time of 2.89 Mya. Clade E1 is found in the eastern North America and Europe with low host-plant specificity. The short-lived North Atlantic Land Bridge acted as a dispersal route until the low Oligocene [6, 36]. Migration to Europe seems unlikely, so the reasonable interpretation is that the human activity introduced this species into new habitats as proposed by Feng et al. [3]. The wind and ocean current could be another driving force and reasonable explanation for the dispersal of fungal basidiospores between Europe and eastern North America [8].

The species in Group VI and Clade A2 have an East Asian-European dispersal route. This route is probable because an exchange of species occurs for *Laetiporus* and its most common host plants such as *Quercus*, *Salix*, *Populus*, *Picea*, *Abies* and *Larix* [1, 22–25, 38]. It is reasonable to accept this route because the Eurasian Plate is continuous.

Group IV consists of three different types of *L. versisporus* that are endemic in East Asia (Figs. 1 and 4). The infraspecific variation is obvious in these three types, but gene exchange and recombination still exist according to the clonal research of Ota et al. [13]. This finding indicates that vicariance is important for regional speciation.

In addition, the migration of Clade I to Hawaii is surprising and worth exploring. We speculate that this example results from an incomplete sampling of molecular data. However, there are many standalone islands in the South Pacific indirectly connecting Hawaii, Australia and Malay Archipelago. The frequent strong winds and continuous ocean currents are potentially responsible for the dispersal of basidiospores between islands. The humid climate and abundant host plants such as *Quercus*, *Castanea* and *Eucalyptus* from the Malay Archipelago to Australia [39, 40] are suitable for *Laetiporus*. A dispersal route of East Asia–Malay Archipelago–Australia–Hawaii seems unlikely. Interestingly, *Eucalyptus*, the host plant of Clade I has been proven to colonize Hawaii via this route [2].

In our study, the samples of *Laetiporus* are scanty in some areas around the world, such as South America, Indian Subcontinent, South Africa and Australia. The taxonomic situation is still unclear, and the evolutionary history of *Laetiporus* remains incompletely understood. A wider range of sampling and further morphological studies, incompatibility tests, and more information of host range and distribution are needed.

## Conclusion

The evolutionary history of *Laetiporus* remains incompletely understood. However, this study presents some progress on this topic. (1) Two novel phylogenetic species in East Asia were identified. (2) Our reconstruction and analysis of ancestral areas suggest that *Laetiporus* originated during the early Miocene (20.16 ± 0.13 Mya) in temperate zones and that the combination of East Asia and North America has the highest probability (48%) of being the ancestral area. (3) We also predict that *Laetiporus* may be present in the Indian Subcontinent, in Australia and in the Malay Archipelago. (4) Four intercontinental dispersal routes and a possible concealed dispersal route are proposed. (5) Vicariance is suggested to play an important role in regional speciation, and recent human activity may render some geographical distribution inexplicable. Further sampling and more molecular data are needed to further clarify the species affinity.

## Methods

### Taxon sampling

**Fig. 6:**
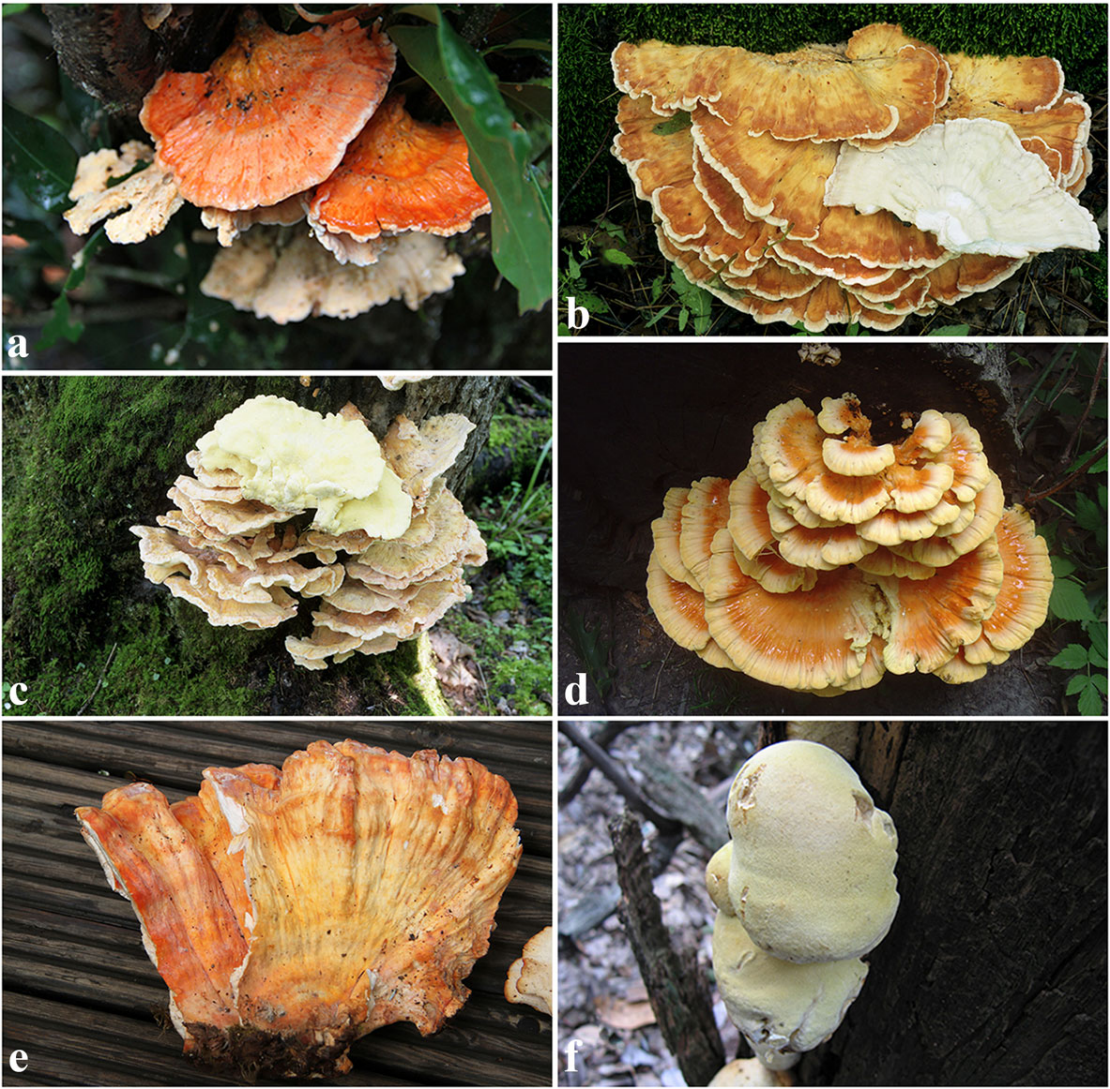
Basidiomata of *Laetiporus* species. **a**–*L. ailaoshanensis*. **b**–*L. cremeiporus.* **c**–*L. montanus*. **d**–*L. sulphurous*. **e**–*L. zonatus*. **f**–*L. versisporus*

This study included 105 samples of *Laetiporus* from East Asia, Europe, North America, Central America, South America, Hawaii and South Africa. Basidiomata of several *Laetiporus* species were shown in Fig. 6. The sequences of the samples obtained for this study were deposited in the herbaria of the Institute of Microbiology, Beijing Forestry University (BJFC), Institute of Microbiology, Chinese Academy of Sciences (HMAS), and Institute of Applied Ecology, Chinese Academy of Sciences (IFP). Each specimen’s scientific name, GenBank accession numbers and other relevant information are listed in Additional file 1: Table S2.

### DNA extraction, PCR, and DNA sequencing

Genomic DNA was extracted from dried fruiting bodies using a cetyltrimethylammonium bromide rapid plant genome extraction kit (Aidlab Biotechnologies Co., Ltd., Beijing) according to the manufacturer’s instructions with some modifications [26]. Six DNA gene fragments were analyzed, including those coding for RPB2 and EF-1α, along with four non-protein coding regions: ITS, nrLSU, nrSSU and mtSSU. These fragments were actually appropriate in determining the taxonomic status of *Laetiporus*. The primer pairs ITS5/4 [29], LR0R/LR7 [41], PNS1/NS41 [42], MS1/MS2 [29], and 983F/1567R [43] were used to amplify ITS, nrLSU, nrSSU, mtSSU and EF-1α, respectively. Initial attempts to amplify RPB2 using previously published primers that were designed for fungi [44] resulted in weak or non-specific amplification. To improve the success rate of RPB2 amplification, a new primer pair, 6F-1 (CCTCGTCAACTGCACAACA) and 7R-1 (TCTTCCTCGGCATCCAA), was designed based on eleven obtained sequences using Primer-Premier 5 (Premier Biosoft International, Palo Alto, CA, USA).

PCR was performed in a reaction mixture containing 25 μl of 2 × EasyTaq*®* PCR SuperMix, 2 μl of Forward Primer (10 μM), 2 μl of Reverse Primer (10 μM), and 2 μl of Template DNA. The total volume was adjusted to 50 μl with sterile deionized H_2_O. The PCR amplifications were conducted using an Eppendorf Master Cycler (Eppendorf, Netheler-Hinz, Hamburg, Germany), and the cycling conditions were follows: pre-denaturation at 95 °C for 4 min; 35 cycles of denaturation at 94 °C for 40 s, annealing at 50 °C–54 °C for 45 s (ITS, mtSSU, EF-1α and RPB2) or for 60 s (nrLSU and nrSSU), and elongation at 72 °C for 60 s (ITS, mtSSU, EF-1α and RPB2) or for 90 s (nrLSU and nrSSU); and a final elongation at 72 °C for 10 min. The PCR products were visualized by agarose gel electrophoresis and stored at −20 °C after visualization. The PCR products were purified and sequenced at the Beijing Genomics Institute (China) using the same primers as those used for amplification. Of the 370 sequences of *Laetiporus* used in this paper, 226 sequences of *Laetiporus* were newly generated, including 28 ITS (27% new), 27 nrLSU (40% new), 41 nrSSU (100% new), 47 mtSSU (78% new), 46 EF-1α (85% new), and 37 RPB2 (88% new). All newly generated sequences were deposited in the GenBank database.

### Sequence alignments and phylogenetic analyses

To determine the phylogeny of *Laetiporus*, we compiled two datasets: the ITS sequences matrix and a concatenated dataset (ITS + nrLSU + nrSSU + mtSSU + EF-1α + RPB2). In the combined dataset, *Antrodia serialis* (Fr.) Donk and *Fomitopsis pinicola* (Sw.) P. Karst. were used as outgroups; the sequences of ITS, nrLSU, nrSSU, mtSSU, EF-1α and RPB2 were aligned initially by using MAFFT 6 [45] using the “G-INS-I” strategy and then manually optimized in BioEdit [46]. Ambiguously aligned regions were excluded from subsequent analyses. Finally, the six gene fragments were concatenated with SEAVIEW 4 [47] for further phylogenetic analyses. One thousand partition homogeneity test (PHT) replicates of the ITS, nrLSU, nrSSU, mtSSU, EF-1α and RPB2 sequences were tested using PAUP* version 4.0b10 [48] to determine whether the partitions were homogeneous. The PHT results indicated that all the DNA sequences had a congruent phylogenetic signal (*P* value =0.19). The ITS dataset included more samples compared to the combined dataset. It contained 100 sequences, of which 98 were *Laetiporus* sequences; *Wolfiporia dilatohypha* Ryvarden & Gilb. was used as an outgroup. The sequences were aligned using the same method as that used for the combined dataset. Sequence alignments were deposited at TreeBase (submission ID 20418, 20,419; www.treebase.org).

ML analysis was conducted using RAxML-HPC2 [49] on Abe through the Cipres Science Gateway [50]. To estimate the branch support with an alternative method, we performed BI and MP analyses. For the ML and BI analyses, the optimal substitution models for ITS and the combined dataset were determined using the Akaike information criterion (AIC) as implemented in MrModeltest v2.3 [51, 52]. The selected substitution models for both the combined dataset and ITS dataset were general time reversible + proportion invariant + gamma (GTR + I + G).

In the ML analysis, the concatenated dataset was partitioned into six parts by sequence region, and 1000 ML searches were run under the GTR + GAMMA model with all model parameters estimated using the RAxML-HPC2 program. The best fit maximum likelihood tree from all searches was kept. In addition, 1000 rapid bootstrap replicates were run with the GTR + CAT model to assess the reliability of the nodes.

BI was performed using MrBayes 3.1.2 [53] with 2 independent runs, each beginning from random trees with 4 simultaneous independent chains, performing 5,000,000 replicates each for the concatenated dataset and the ITS dataset, sampling one tree every 1000 generations. Chain convergence was determined using Tracer v1.5 (http://tree.bio.ed.ac.uk/software/tracer/) to confirm sufficiently large ESS values (>200). The first 25% of the sampled trees were discarded as burn-in, and the remaining trees were used to reconstruct a majority rule consensus and calculate BPP of the clades.

MP analysis was performed in PAUP* version 4.0b10 [48]. All characters were equally weighted, and gaps were treated as missing data. Trees were inferred using the heuristic search option with TBR branch swapping and 1000 random sequence additions. Max-trees were set to 5000, branches of zero length were collapsed, and all parsimonious trees were saved. Clade robustness was assessed using a bootstrap (BT) analysis with 1000 replicates [54].

Branches of the consensus tree that received bootstrap support for MP, ML and BPP greater than or equal to 75% (MP/ML) and 0.95 (BPP) were considered to be significantly supported.

### Molecular dating analysis

Given that fossil records of fungi are limited, it is difficult to choose a reliable calibration point to estimate the divergence time for any fungal groups. Therefore, extensive sampling of outgroup species for which fossils were available was performed in order to estimate the divergence time of *Laetiporus*. Two primary calibration points were included in our analyses: (1) the divergence between Ascomycota and Basidiomycota, 582 Mya, by placing *P. devonicus* in the subphylum Pezizomycotina [55]; and (2) the divergence between Hymenochaetaceae and Fomitopsidaceae based on a 125 million-year-old fossil of *Q. cranhamii* [56]. The parameter settings for the two calibrations were the same as those used in Feng et al. [3]. As the identifications of the two fossils were fairly ambiguous, the estimated divergence time was constrained by the following two values: the estimated divergence time between Ascomycota and Basidiomycota is at least 400 Mya (the divergence time of *P. devonicus*), and the initial diversification of *Laetiporus* should be close to the divergence times of their host plants as suggested by the co-evolution of the fungi and the plants [57]. The calibration point for which the estimated results met these two criteria was eventually chosen for our subsequent analyses.

Three nuclear ribosomal RNA genes (ITS, nrLSU and nrSSU) and two protein coding genes (EF-1α and RPB2) were concatenated for molecular dating using the phylogenetic framework described in James et al. [58]. ITS1, ITS2, and the introns in EF-1α and RPB2 were excluded for a conservation analysis. All of the outgroup sequences were retrieved from GenBank and are listed in Additional file 1: Table S2. MrModeltest v2.3 [51, 52] was used to select the best models of evolution using the hierarchical likelihood ratio test. The selected evolutionary models for the two combined datasets were GTR + I + G. The origin time of *Laetiporus* was estimated in BEAST v1.8.0 [59] with the molecular clock and substitution models unlinked but with the trees linked for each gene partition. The BEAST input files were constructed using BEAUti (within BEAST). The lognormal relaxed molecular clock and the Yule speciation prior set were used to estimate the divergence time and the corresponding credibility intervals. The posterior distributions of parameters were obtained using MCMC analysis for 50 million generations with a burn-in percentage of 10%. The convergence of the chains was confirmed using Tracer v1.6. Samples from the posterior distributions were summarized on a maximum clade credibility tree with the maximum sum of posterior probabilities listed on its internal nodes using TreeAnnotator v1.8.0 [59] with the posterior probability limits set to 0.5 to summarize the mean node heights. FigTree v1.4.2 [60] was used to visualize the resulting tree and to obtain the means and 95% HPD [59]. A 95% HPD marks the shortest interval that contains 95% of the values sampled.

We also estimated the divergence time of the main nodes in *Laetiporus* using the ITS dataset, which contained one or two representatives of all of the *Laetiporus* species that were included in our analyses. The estimated crown age of *Laetiporus* based on the combined ITS + nrLSU + nrSSU and EF-1α + RPB2 datasets was used as the calibration point to date the ITS phylogeny by setting the prior to a normal distribution. The other procedures were the same as those applied in the estimation using the combined dataset.

### Biogeographic analysis

The reconstruction of ancestral areas in a phylogeny is important for understanding the biogeographic diversification history of a lineage, as this reconstruction makes it possible to infer the original location and dispersal routes of the organisms. To infer ancestral areas, the DEC [61] model was used in RASP 3.2 [62], allowing a maximum of two areas per node. The ancestral area analyses were conducted using the posterior distributions of the dated ITS phylogeny that were estimated from BEAST. The geographic distributions for the *Laetiporus* were delimited into seven areas: (A) East Asia, (B) Europe, (C) North America, (D) Central America, (E) South America, (F) South Africa, and (G) Hawaii. ArcGIS v10.1 [63] was used to visualize the geographic distribution and possible dispersal routes of *Laetiporus*.

## Additional file

Additional file 1: Table S1. Estimated divergence times of the main nodes correspond with the dating analysis of ITS + nrLSU + nrSSU and EF-1α + RPB2 datasets. **Table S2.** Information about the samples used in this study. **Table S3.** Estimated divergence times of the main groups correspond with the dating analysis of ITS datasets. (DOCX 56 kb)

## Abbreviations

BI: Bayesian inference
BJFC: Herbaria of the Institute of Microbiology, Beijing Forestry University
BPP: Bayesian posterior probabilities
DEC: Dispersal-Extinction-Cladogenesis
EF-1α: Translation elongation factor 1-α
GTR + I + G: General time reversible + proportion invariant + gamma
HMAS: Institute of Microbiology, Chinese Academy of Sciences
HPD: Higher posterior densities
IFP: Institute of Applied Ecology, Chinese Academy of Sciences
ITS: Internal transcribed spacer
ML: Maximum likelihood
MP: Maximum parsimony
mtSSU: Mitochondrial small subunit rDNA
Mya: Million years ago
nrLSU: Nuclear large subunit rDNA
nrSSU: Nuclear small subunit rDNA
RPB2: DNA-directed RNA polymerase II subunit 2
