# Supplementary material for: Phylogeny, divergence time and historical biogeography of Laetiporus (Basidiomycota, Polyporales)
Source: BMC Evol Biol. 2017 Apr 20;17:102. doi: 10.1186/s12862-017-0948-5 (PMC5397748; doi:10.1186/s12862-017-0948-5)
Supplement: Additional file 1: Table S1. — Estimated divergence times of the main nodes correspond with the dating analysis of ITS + nrLSU + nrSSU and EF-1α + RPB2 datasets. Table S2. Information about the samples used in this study. Table S3. Estimated divergence times of the main groups correspond with the dating analysis of ITS datasets. (DOCX 56 kb) [file 12862_2017_948_MOESM1_ESM.docx]

**Table**

**Table 1.** Estimated divergence times of the main nodes correspond with the dating analysis of ITS+nrLSU+nrSSU and EF-1α+RPB2 datasets.

| Node | Mean ± standard error | 95% HPD | Node | Mean ± standard error | 95% HPD |
| --- | --- | --- | --- | --- | --- |
| A: Ascomycota/Basidiomycota | 564.05 ± 0.35 | 464.44–664.77 | G: Gomphales/Agaricomycetes | 269.59 ± 1.01 | 200.71–339.56 |
| B: Pucciniomycitina/Basidiomycota | 497.68 ± 0.77 | 393.44–601.89 | H: Russulales/Agaricomycetes | 246.72 ± 1.00 | 183.67–311.49 |
| C: Ustilaginomycotina/Agaricomycotina | 485.81 ± 0.78 | 383.44–401.39 | I: Agaricales/Agaricomycetes | 233.61 ± 0.98 | 175.37–297.36 |
| D: Tremellomycetes/Agaricomycotina | 433.52 ± 0.87 | 335.60–533.36 | J: Marasmius, Mycena/Agaricomycetes | 225.31 ± 0.96 | 167.92–286.10 |
| E: Dacrymycetes/Agaricomycetes | 392.56 ± 0.95 | 300.00–487.96 | K: Polyporales | 194.56 ± 0.89 | 141.93–247.52 |
| F: Hymenochaetales/Agaricomycetes | 328.00 ± 1.15 | 245.47–415.08 | L: *Laetiporus* | 20.17 ± 0.12 | 12.66–29.09 |

**Table 2.** Information of the samples used in this study.

| **Species** | **Herbarium** | **Collection no.** | **GenBank Accessions** | | | | | |
| --- | --- | --- | --- | --- | --- | --- | --- | --- |
|  |  |  | **ITS/5.8s** | **nrLSU** | **nuSSU** | **mtSSU** | **EF-1α** | **RPB2** |
| *Agaricostilbum hyphaenes* | – | AFTOL 675 | AY789077 | AY634278 | AY665775 | – | AY879114 | AY780933 |
| *Antrodia serialis* | BJFC | Cui 10519 | KP715307 | KP715323 | KR605911 | KR606011^a^ | KP715337 | KR610830^a^ |
| *Calocera cornea* | – | AFTOL 438 | AY789083 | AY701526 | AY771610 | – | AY881019 | AY536286 |
| *Clavaria zollingeri* | – | AFTOL 563 | AY854071 | AY639882 | AY657008 | – | AY881024 | AY780940 |
| *Coltricia perennis* | – | AFTOL 447 | DQ234559 | AF287854 | U59064 | – | AY885147 | AY218526 |
| *Cryptococcus humicola* | – | AFTOL 1552 | DQ645516 | DQ645514 | DQ645515 | – | DQ645519 | DQ645517 |
| *Dacryopinax spathularia* | – | AFTOL 454 | AY854070 | AY701525 | AY771603 | – | AY881020 | AY786054 |
| *Daedalea quercina* | BJFC | Dai 12152 | KP171207 | KP171229 | KR605886 | – | KR610717 | KR610809^a^ |
| *Echinodontium tinctorium* | – | AFTOL 455 | AY854088 | AF393056 | U59068 | – | AY885157 | AY218482 |
| *Fomitiporia hartigii* | MUCL | MUCL 53551 | JX093789 | JX093833 | KX357140^a^ | – | JX093746 | JX093877 |
| *F. mediterranea* | – | AFTOL 688 | AY854080 | AY684157 | AY662664 | – | AY885149 | AY803748 |
| *Fomitopsis betulinus* | BJFC | Dai 12665 | KP171215 | KP171238 | KR605896 | – | KR610724 | KR610817^a^ |
| *F. pinicola* | BJFC | Cui 10405 | KC844852 | KC844857 | KR605857 | KR605961^a^ | KR610690 | KR610781^a^ |
| *Gautieria otthii* | – | AFTOL 466 | AY883434 | AF393058 | AF393043 | – | AY883434 | AY218486 |
| *Heterobasidion annosum* | BJFC | 06129/6 | KJ583211 | KJ583225 | U59072 | – | KX252741^a^ | KF033133 |
| *Hygrocybe conica* | – | AFTOL 729 | AY854074 | AY684167 | AY752965 | – | AY883425 | AY803747 |
| *Lactarius deceptivus* | – | AFTOL 682 | AY854089 | AY631899 | AY707093 | – | AY885158 | AY803749 |
| *Laetiporus ailaoshanensis* | BJFC | Dai 15624 | KX354467^a^ | KX354495^a^ | KX354532^a^ | KX354574^a^ | KX354620^a^ | KX354663^a^ |
| *L. ailaoshanensis* | BJFC | Dai 13574 | KX354468^a^ | KX354496^a^ | KX354533^a^ | KX354575^a^ | KX354621^a^ | KT894794 |
| *L. ailaoshanensis* | BJFC | Cui 12387 | KX354469^a^ | KX354497^a^ | KX354534^a^ | KX354576^a^ | KX354622^a^ | KX354664^a^ |
| *L. ailaoshanensis* | BJFC | Dai 13567 | KX354470^a^ | KX354498^a^ | KX354535^a^ | KX354577^a^ | KX354623^a^ | KX354665^a^ |
| *L. ailaoshanensis* | BJFC | Dai 13566 | KX354471^a^ | KX354499^a^ | KX354536^a^ | KX354578^a^ | KX354624^a^ | KX354666^a^ |
| *L. ailaoshanensis* | BJFC | Dai 13256 | KF951289 | KF951317 | KX354537^a^ | KX354579^a^ | KX354625^a^ | KT894786 |
| *L. caribensis* | CFMR | PR 6583 | JN684766 | – | – | – | – | – |
| *L. caribensis* | CFMR | GDL 1 | EU402547 | EU402525 | – | EU402483 | – | – |
| *L. caribensis* | CFMR | PR 914 | JN684762 | EU402526 | – | EU402482 | – | – |
| *L. caribensis* | CFMR | PR 6521 | JN684771 | – | – | – | – | – |
| *L. cincinnatus* | BJFC | Dai 12811 | KF951291 | KF951304 | KX354516^a^ | KX354558^a^ | KX354605^a^ | KT894788 |
| *L. cincinnatus* | CFMR | DA 37 | EU402557 | EU402521 | – | EU402485 | AB472661 | – |
| *L. cincinnatus* | BJFC | JV 0709/168J | KF951290 | KF951305 | KX354517^a^ | KX354559^a^ | KX354606^a^ | KX354651^a^ |
| *L. cincinnatus* | CFMR | 46-1104 | EU402560 | – | – | – | – | – |
| *L. conifericola* | BJFC | JV 0709/81J | KF951292 | KF951327 | KX354531^a^ | KX354573^a^ | – | KX354683^a^ |
| *L. conifericola* | CFMR | CA 8 | EU402575 | EU402523 | – | EU402487 | AB472663 | – |
| *L. conifericola* | CFMR | JAM 1 | EU402577 | EU402524 | – | EU402486 | AB472664 | – |
| *L. conifericola* | CFMR | NV 2 | AB472633 | – | – | – | – | – |
| *L. cremeiporus* | BJFC | Cui 10586 | KF951277 | KF951297 | KX354513^a^ | KX354555^a^ | KX354602^a^ | KX354648^a^ |
| *L. cremeiporus* | BJFC | Li 140927 | KX354459^a^ | KX354485^a^ | KX354514^a^ | KX354556^a^ | KX354603^a^ | KX354649^a^ |
| *L. cremeiporus* | BJFC | Dai 10107 | KF951281 | KF951301 | KX354515^a^ | KX354557^a^ | KX354604^a^ | KX354650^a^ |
| *L. cremeiporus* | BJFC | Cui 10991 | KF951279 | KF951298 | – | KX354595^a^ | KX354641^a^ | KX354679^a^ |
| *L. cremeiporus* | – | 11208A | EU840623 | – | – | – | – | – |
| *L. cremeiporus* | – | JR040721-24 | EU840628 | – | – | – | – | – |
| *L. cremeiporus* | – | KR960611-13 | EU840624 | – | – | – | – | – |
| *L. cremeiporus* | FFPRI | WD 2307 | AB308178 | – | – | – | – | – |
| *L. cremeiporus* | FFPRI | WD 2306 | AB308177 | – | – | – | – | – |
| *L. gilbertsonii* | BJFC | JV 1109/31 | KF951293 | KF951306 | KX354542^a^ | KX354584^a^ | KX354630^a^ | KX354671^a^ |
| *L. gilbertsonii* | CFMR | CA 13 | EU402549 | EU402527 | – | EU402496 | AB472666 |  |
| *L. gilbertsonii* | BJFC | TJV 2000/101 | EU402553 | EU402528 | – | EU402493 | AB472668 |  |
| *L. gilbertsonii* | BRH | BZ 544 | JN684768 | – | – | – | – | – |
| *L. gilbertsonii* | CFMR | Lowe 12954 | JN684767 | – | – | – | – | – |
| *L. gilbertsonii* | CFMR | FP 150268 | EU402552 | – | – | – | – | – |
| *L. gilbertsonii* | – | 5032 | EU840668 | – | – | – | – | – |
| *L. gilbertsonii* | SP | CCIBt 542 | KP765241 | – | – | – | – | – |
| *L. gilbertsonii* | – | 5067 | EU840670 | – | – | – | – | – |
| *L. gilbertsonii* | CORD | Robledo 47 | JN684769 | – | – | – | – | – |
| *L. huroniensis* | CFMR | HMC 3 | EU402571 | EU402540 | – | – | – | – |
| *L. huroniensis* | CFMR | MI 14 | EU402573 | EU402539 | – | EU402489 | AB472672 | – |
| *L. montanus* | BJFC | Cui 10011 | KF951274 | KF951315 | KX354528^a^ | KX354570^a^ | KX354617^a^ | KT894790 |
| *L. montanus* | BJFC | Dai 15888 | KX354466^a^ | KX354494^a^ | KX354530^a^ | KX354572^a^ | KX354619^a^ | KX354662^a^ |
| *L. montanus* | BJFC | Cui 10015 | KF951273 | KF951311 | KX354529^a^ | KX354571^a^ | KX354618^a^ | KT894791 |
| *L. montanus* | – | L12-706688 | EU840558 | – | – | – | – | – |
| *L. montanus* | IRZ | L 2 | AB472614 | – | – | – | – | – |
| *L. montanus* | IRZ | L 30 | AB472617 | – | – | – | – | – |
| *L. montanus* | FFPRI | WD 2303 | AB308194 | – | – | – | – | – |
| *L.* sp. 1 | CFMR | EUC 1 | EU402545 | EU402541 | – | – | – | – |
| *L.* sp. 1 | CFMR | KOA 1 | EU402546 | EU402542 | – | – | – | – |
| *L.* sp. 2 | – | RV2A | EU840664 | – | – | – | – | – |
| *L.* sp. 2 | – | RV3A | EU840665 | – | – | – | – | – |
| *L.* sp. 2 | – | RV4A | EU840662 | – | – | – | – | – |
| *L.* sp. 2 | – | RV5A | EU840663 | – | – | – | – | – |
| *L.* sp. 3 | BJFC | Cui 12219 | KX354472^a^ | KX354500^a^ | KX354538^a^ | KX354580^a^ | KX354626^a^ | KX354667^a^ |
| *L.* sp. 3 | BJFC | Cui 12240 | KX354473^a^ | KX354501^a^ | KX354539^a^ | KX354581^a^ | KX354627^a^ | KX354668^a^ |
| *L.* sp. 3 | BJFC | Cui 12390 | KX354474^a^ | KX354502^a^ | KX354540^a^ | KX354582^a^ | KX354628^a^ | KX354669^a^ |
| *L.* sp. 4 | BJFC | Dai 15953 | KX354460^a^ | KX354488^a^ | KX354522^a^ | KX354564^a^ | KX354611^a^ | KX354656^a^ |
| *L.* sp. 4 | BJFC | Dai 15828 | KX354461^a^ | KX354489^a^ | KX354523^a^ | KX354565^a^ | KX354612^a^ | KX354657^a^ |
| *L.* sp. 4 | BJFC | Dai 15905 | KX354462^a^ | KX354490^a^ | KX354524^a^ | KX354566^a^ | KX354613^a^ | KX354658^a^ |
| *L.* sp. 4 | BJFC | Dai 15902 | KX354463^a^ | KX354491^a^ | KX354525^a^ | KX354567^a^ | KX354614^a^ | KX354659^a^ |
| *L.* sp. 4 | BJFC | Dai 15898A | KX354464^a^ | KX354492^a^ | KX354526^a^ | KX354568^a^ | KX354615^a^ | KX354660^a^ |
| *L.* sp. 4 | BJFC | Dai 15825 | KX354465^a^ | KX354493^a^ | KX354527^a^ | KX354569^a^ | KX354616^a^ | KX354661^a^ |
| *L.* sp. 5 | CFMR | Munez 207 | JN684764 | – | – | – | – | – |
| *L.* sp. 6 | CORD | Robledo 1122 | JN684765 | – | – | – | – | – |
| *L. sulphureus*-1 | BJFC | Cui 12389 | KR187106^a^ | KX354487^a^ | KX354519^a^ | KX354561^a^ | KX354608^a^ | KX354653^a^ |
| *L. sulphureus*-1 | BJFC | Cui 12388 | KR187105^a^ | KX354486^a^ | KX354518^a^ | KX354560^a^ | KX354607^a^ | KX354652^a^ |
| *L. sulphureus*-1 | BJFC | JV 1106/15 | KF951296 | KF951303 | KX354520^a^ | KX354562^a^ | KX354609^a^ | KX354654^a^ |
| *L. sulphureus*-1 | BJFC | Dai 12154 | KF951295 | KF951302 | KX354521^a^ | KX354563^a^ | KX354610^a^ | KX354655^a^ |
| *L. sulphureus*-1 | MUCL | MUCL 8890 | AB472605 | – | – | – | – | – |
| *L. sulphureus*-1 | IRZ | L 7 | AB472613 | – | – | – | – | – |
| *L. sulphureus*-1 | IRZ | 940519.1M1A | AB472610 | – | – | – | – | – |
| *L. sulphureus*-1 | – | L14-706686 | EU840555 | – | – | – | – | – |
| *L. sulphureus*-1 | MUCL | MUCL 34165 | AB472608 | – | – | – | – | – |
| *L. sulphureus*-2 | BJFC | Cui 12370 | KX354477^a^ | KX354504^a^ | KX354543^a^ | KX354585^a^ | KX354631^a^ | KX354672^a^ |
| *L. sulphureus*-2 | BJFC | Cui 12371 | KX354478^a^ | KX354505^a^ | KX354544^a^ | KX354586^a^ | KX354632^a^ | KX354673^a^ |
| *L. sulphureus*-2 | BJFC | Z.R.L. CA04 | KX354479^a^ | KX354506^a^ | KX354545^a^ | KX354587^a^ | KX354633^a^ | KX354674^a^ |
| *L. sulphureus*-2 | BJFC | Z.R.L. CA08 | KX354480^a^ | KX354507^a^ | KX354546^a^ | KX354588^a^ | KX354634^a^ | KX354675^a^ |
| *L. sulphureus*-2 | CFMR | GR 12 | EU402561 | EU402534 | – | EU402480 | AB472658 | – |
| *L. sulphureus*-2 | CFMR | CT 1 | EU402565 | EU402532 | – | EU402479 | AB472659 | – |
| *L. sulphureus*-2 | CFMR | DA 41 | EU40256 | EU402533 | – | EU402481 | AB472660 | – |
| *L. sulphureus*-2 | – | VITTOR-SP | EU840600 | – | – | – | – | – |
| *L. sulphureus*-2 | – | Q2-CZ | EU840563 | – | – | – | – | – |
| *L. sulphureus*-2 | – | 6730 | EU840681 | – | – | – | – | – |
| *L. sulphureus*-2 | – | OLRIM 1025 | EU840566 | – | – | – | – | – |
| *L. sulphureus*-3 | BJFC | TJV 99/150 | EU402567 | EU402530 | – | EU402492 | – | – |
| *L. sulphureus*-3 | CFMR | MAS 2 | EU402568 | EU402531 | – | EU402491 | – | – |
| *L. versisporus*-1 | BJFC | Cui 7882 | KF951269 | KF951323 | – | KX354596^a^ | KX354642^a^ | KT894783 |
| *L. versisporus*-1 | BJFC | Dai 13160 | KF951266 | KF951320 | – | KX354597^a^ | KX354643^a^ | KT894785 |
| *L. versisporus*-1 | BJFC | Li 15071314 | KX354476^a^ | KX357139^a^ | – | KX354598^a^ | KX354644^a^ | KX354680^a^ |
| *L. versisporus*-1 | BJFC | Cui 9154 | KF951270 | KF951322 | – | KX354599^a^ | KX354645^a^ | KT894784 |
| *L. versisporus*-1 | BJFC | Cui 5488 | KF951268 | KF951321 | – | – | – | – |
| *L. versisporus*-1 | FFPRI | WD 820 | AB308135 | – | – | – | – | – |
| *L. versisporus*-2 | IFP | Yuan 6319 | KX354475^a^ | KX354503^a^ | KX354541^a^ | KX354583^a^ | KX354629^a^ | KX354670^a^ |
| *L. versisporus*-2 | – | 3296G | EU840625 | – | – | – | – | – |
| *L. versisporus*-2 | – | 7133A | EU840626 | – | – | – | – | – |
| *L. versisporus*-2 | FFPRI | WD 2437 | AB308141 | – | – | – | – | – |
| *L. versisporus*-2 | FFPRI | L 140 | AB472592 | – | – | – | – | – |
| *L. versisporus*-3 | BJFC | Dai 10992 | KF951272 | KF951325 | – | KX354600^a^ | KX354646^a^ | KX354681^a^ |
| *L. versisporus*-3 | BJFC | Dai 13052 | KF951271 | KF951324 | – | KX354601^a^ | KX354647^a^ | KX354682^a^ |
| *L. zonatus* | BJFC | Dai 13633 | KX354481^a^ | KX354508^a^ | KX354547^a^ | KX354589^a^ | KX354635^a^ | KX354676^a^ |
| *L. zonatus* | HKAS | HKAS 71806 | KF951284 | KF951310 | KX354548^a^ | KX354590^a^ | KX354636^a^ | KT894796 |
| *L. zonatus* | BJFC | SAAS 547 | KX354482^a^ | KX354509^a^ | KX354549^a^ | KX354591^a^ | KX354637^a^ | KX354677^a^ |
| *L. zonatus* | BJFC | Cui 10403 | KF951282 | KF951307 | KX354550^a^ | KX354592^a^ | KX354638^a^ | – |
| *L. zonatus* | BJFC | Cui 10404 | KF951283 | KF951308 | KX354551^a^ | KX354593^a^ | KX354639^a^ | KT894797 |
| *L. zonatus* | BJFC | SAAS 681 | KX354483^a^ | KX354510^a^ | KX354552^a^ | KX354594^a^ | KX354640^a^ | KX354678^a^ |
| *L. zonatus* | BJFC | Cui 12499 | KX354484^a^ | – | – | – | – | – |
| *Mycena amabilissima* | – | AFTOL 1685 | DQ490646 | DQ470811 | DQ457694 | – | GU187728 | DQ474122 |
| *M. aurantiidisca* | – | AFTOL 1686 | DQ490644 | DQ457691 | DQ457647 | – | GU187727 | DQ474121 |
| *Marasmius rotula* | – | AFTOL 1505 | DQ182506 | DQ457686 | DQ113912 | – | GU187723 | DQ474118 |
| *Neurospora crassa* | – |  | HQ271348 | AF286411 | AY046271 | – | XM_959775 | AF107789 |
| *Phaeolus schweinitzii* | BJFC | Dai 8025 | KX354457^a^ | KX354511^a^ | KX354553^a^ | – | KX354686^a^ | DQ408119 |
| *Polyporus squamosus* | – | AFTOL 704 | DQ267123 | AY629320 | AY705963 | – | DQ028601 | DQ408120 |
| *Pycnoporellus fulgens* | BJFC | Cui 10033 | KX354458^a^ | KX354512^a^ | KX354554^a^ | – | KX354687^a^ | KX354684^a^ |
| *Ramaria rubella* | – | AFTOL 724 | AY854078 | AY645057 | AY707095 | – | AY883435 | AY786064 |
| *Rhizopus stolonifer* | – | CBS 609.82 | AB113023 | DQ273817 | DQ536474 | – | AB512268 | AFTOL datebase |
| *Schizosaccharomyces pombe* | – | 972h | Z19578 | Z19136 | JN938992 | – | NM_001021161 | NM_001018498 |
| *Sparassis crispa* | – | AFTOL 703 | DQ250597 | AY629321 | AY705962 | – | DQ056289 | DQ408122 |
| *Stereum hirsutum* | – | AFTOL 492 | AY854063 | AF393078 | U59095 | – | AY885159 | AY218520 |
| *Trametes versicolor* | – | FP 135156sp | JN164919 | JN164809 | AY336751 | – | JN164878 | JN164850 |
| *Ustilago maydis* | – |  | AY854090 | AF453938 | X62396 | – | AY885160 | AY485636 |
| *Wolfiporia cocos* | BJFC | CBK 1 | KX354453^a^ | KX354689^a^ | KX354690^a^ | – | KX354688^a^ | KX354685^a^ |
| *W. dilatohypha* | – | CS 63 | EU402555 | – | – | – | – | – |
| *W. dilatohypha* | – | FP 72162 | EU402556 | – | – | – | – | – |

^a^ Newly generated sequences for this study.

**Table 3.** Estimated divergence times of the main groups correspond with the dating analysis of ITS datasets.

| Group | Mean ± standard error | 95% HPD | Group | Mean ± standard error | 95% HPD |
| --- | --- | --- | --- | --- | --- |
| Group I | 4.64 ± 0.04 | 2.30–6.98 | Group II | 9.88 ± 0.05 | 4.95–14.28 |
| Group III | 6.35 ± 0.05 | 2.86–9.84 | Group IV | 6.70 ± 0.05 | 3.02–11.71 |
| Group V | 2.89 ± 0.03 | 1.15–4.63 | Group VI | 1.68 ± 0..01 | 0.98–2.38 |
